# *CCR5* Haplotypes Influence HCV Serostatus in Caucasian Intravenous Drug Users

**DOI:** 10.1371/journal.pone.0070561

**Published:** 2013-07-25

**Authors:** Kristi Huik, Radko Avi, Andrew Carrillo, Nathan Harper, Merit Pauskar, Maarja Sadam, Tõnis Karki, Tõnu Krispin, Ulvi-Kaire Kongo, Tatiana Jermilova, Kristi Rüütel, Ave Talu, Katri Abel-Ollo, Anneli Uusküla, Sunil K. Ahuja, Weijing He, Irja Lutsar

**Affiliations:** 1 Department of Microbiology, Faculty of Medicine, University of Tartu, Tartu, Estonia; 2 Veterans Administration Research Center for AIDS and HIV-1 Infection, and Center for Personalized Medicine, South Texas Veterans Health Care System, San Antonio, Texas, United States of America; 3 Departments of Medicine, University of Texas Health Science Center, San Antonio, Texas, United States of America; 4 Immunoheamatology Reference Laboratory, North Estonia Medical Centre Foundation, Tallinn, Estonia; 5 Blood Center of Kohtla-Järve, Kohtla-Järve, Estonia; 6 National Institute for Health Development, Tallinn, Estonia; 7 Department of Public Health, Faculty of Medicine, University of Tartu, Tartu, Estonia; University of New South Wales, Australia

## Abstract

**Background:**

Up to 90% HIV-1 positive intravenous drug users (IDUs) are co-infected with HCV. Although best recognized for its function as a major co-receptor for cell entry of HIV, CC chemokine receptor 5 (CCR5) has also been implicated in the pathogenesis of HCV infection. Here, we investigated whether *CCR5* haplotypes influence HIV-1 and HCV seropositivity among 373 Caucasian IDUs from Estonia.

**Methods:**

Of these IDUs, 56% and 44% were HIV and HCV seropositive, respectively, and 47% were coinfected. 500 blood donors seronegative for HIV and HCV were also evaluated. *CCR5* haplotypes (HHA to HHG*2) were derived after genotyping nine *CCR2*–*CCR5* polymorphisms. The association between *CCR5* haplotypes with HIV and/or HCV seropositivity was determined using logistic regression analysis. Co-variates included in the models were length of intravenous drug use, HBV serostatus and copy number of *CCL3L1*, the gene encoding the most potent HIV-suppressive chemokine and ligand for CCR5.

**Results:**

Compared to IDUs seronegative for both HCV and HIV (HCV−/HIV-), IDUs who were HCV+/HIV- and HCV+/HIV+were 92% and 82%, respectively, less likely to possess the *CCR5*-HHG*1 haplotype, after controlling for co-variates (P_adjusted_ = 1.89×10^−4^ and 0.003, respectively). This association was mostly due to subjects bearing the *CCR5* HHE and HHG*1 haplotype pairs. Approximately 25% and<10% of HCV−/HIV- IDUs and HCV−/HIV- blood donors, respectively, possessed the HHE/HHG*1 genotype.

**Conclusions:**

Our findings suggest that HHG*1-bearing *CCR5* genotypes influence HCV seropositivity in a group of Caucasian IDUs.

## Introduction

Infection with human immunodeficiency virus 1 (HIV-1) and hepatitis C virus (HCV) remains a source of high morbidity and mortality worldwide [1]–[4]. Among subjects at risk for acquiring HIV or HCV infection [e.g. intravenous drug users (IDU)], co-infection rates can be as high as 90% [2]. Although IDU’s provide an excellent model system to assess genetic risk factors that associate with variable susceptibility to HCV and HIV, the high rates of co-infection make it difficult to distinguish between genetic factors that associate specifically with risk of HIV vs. HCV infection, or both. Consequently, the commonality in the risk factors for acquiring HIV and HCV infection and HIV-HCV co-infection may, depending upon the cohort or population studied, complicate interpretation of genetic association studies [5]. For example, a previous study found that homozygosity for the 32-bp (Δ32) deletion mutation in the coding region of CC chemokine receptor 5 (CCR5), the major co-receptor for cell entry of HIV, was overrepresented in a group of HCV-positive IDUs [6]. It was inferred that the *CCR5-*Δ*32/*Δ*32* mutation is a susceptibility factor for HCV infection. However, because *CCR5-*Δ*32/*Δ*32* mutation associates with strong protection against HIV infection, an alternative explanation could be that HCV-positive survivors in populations under selective pressure of HIV infection (e.g. hemophiliacs), the overrepresentation of *CCR5-*Δ*32/*Δ*32* genotype could represent those who resisted HIV infection [5].

To identify genetic factors that associate with risk of acquiring HCV or HIV, or both concurrently, we recently analyzed a well-characterized group of high-risk IDUs from Estonia, a geographic region that has witnessed an abrupt rise of HIV infection among IDUs since the year 2000 [7]. We found that a high copy number of *CCL3L1*, the gene encoding the most potent HIV suppressive ligand of CCR5, associated with a reduced risk of HIV seropositivity after controlling for HCV and HBV co-infection status as well as length of intravenous drug use (IVDU). An association between *CCL3L1* copy number and HCV or HBV status was not detected. Because extensive data has demonstrated that variations in the non-coding regions (e.g. promoters) of *CCR5* also associate with variable HIV-AIDS susceptibility [8]–[14] and since CCR5 may influence pathogenesis of HCV infection [6], [15]–[24], here, we determined the associations between polymorphisms in *CCR5* and HCV and/or HIV serostatus in this IDU study population from Estonia.

## Materials and Methods

### Subjects and Sample Collection

The IDU population studied was described previously [7]. Briefly, we recruited 373 Caucasian IDUs in 2006 and 2007 from syringe-exchange programs (n = 270) using a respondent-driven sampling [25], [26] and from three Estonian prisons (n = 103). There were 300 males, 55 females and 18 with gender unknown. The median age in the overall IDUs was 26 years (interquartile range, IQR: 22–29 years). All study subjects were Caucasians from Estonia. Altogether seven subjects (3.4%) reported that they had received or were receiving antiretroviral therapy.

The demographics of subjects from syringe-exchange programs and from prisons were similar in terms of age and gender (p>0.1). 92% of the subjects from the syringe-exchange program recorded duration of IVDU but these data were not available in prison subjects due to technical errors. Other risk behavior data (e.g. MSMs and heterosexual contacts) were not available. It was not feasible to recruit HIV-negative or HIV and HCV negative subjects from prisons. There is a high rate of HIV infection as well as HIV/HCV coinfection rate in the subjects from prison. Prisoners indicated their previous drug use in questionnaires as according to official sources there is no drug use inside the prisons.

A control group of anonymous 500 subjects seronegative for HIV-1, HCV and HBV were recruited in 2010 from Caucasian blood donors in the same geographic area from which the IDU study participants were derived. Specially trained nurses informed all IDU participants who then voluntarily signed the consent form. All blood donors signed a form of agreement to use their leftover blood samples for research purposes. The written consent of these healthy donors is not required since no subject characteristics were collected and there is no possibility to identify them. Ethics Committees of Tallinn, University of Tartu and University of Texas Health Science Center at San Antonio approved these studies.

### Laboratory Analyses and Genotyping

HIV, HCV and HBV serostatus was determined at the Estonian HIV Reference Laboratory. HCV and HBV antibodies were assessed by ETI-AB-HCVK-3 anti-HCV test (DiaSorin, Vercelli, Italy), ETI-MAK-4 HBsAg (DiaSorin, Vercelli, Italy), and ETIAB-COREK Plus (anti-HBc core) (DiaSorin, Vercelli, Italy) assays. HIV testing was performed by using a fourth generation enzyme-linked immunoassay (Vironistica HIV Uniform II Ag/Ab, BioMerieux, Marcy Etoile, France) and confirmed by immunoblotting (INNO LIA HIV I/II Score Westernblot (Microgen Bioproducts Ltd, Surrey, UK). Persons were considered HBV positive if they had seropositivity for Core antibody (anti-HBcAb) or surface antigen (anti-HBsAg). The nationwide immunization program against HBV was initiated in 1999 to adolescence aged 12–13 years but the vaccination rates in 1999–2000 did not exceed 40%. More than 80% of our studied IDUs were older than that age at that time. Therefore, the rate of HBV vaccination in our studied IDUs was estimated to be very low thus unlikely influenced the study results.

**Figure 1:**
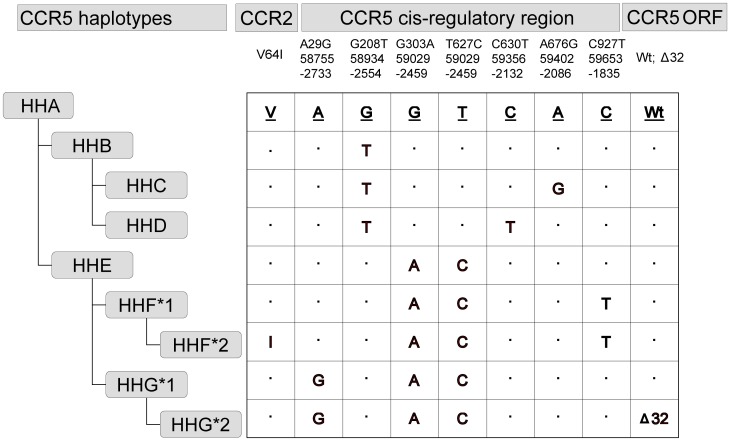
*CCR5* polymorphisms and haplotypes. On the basis of the linkage disequilibrium patterns between the polymorphisms in the coding (Δ32) and noncoding (promoter) region of *CCR5* and the coding polymorphism (V64I) in *CCR2*, we previously used an evolutionary-based strategy to generate the *CCR5* human haplogroups (HH) shown below the CCR5 gene structure. These *CCR5* HH are designated as HHA to HHG*2, with HHF*2 and HHG*2 denoting the haplotypes that bear the CCR2–64I and CCR5-Δ32 polymorphisms, respectively. Because of its similarity to the chimpanzee *CCR5* sequence, the human *CCR5* HHA haplotype is classified as the ancestral CCR5 haplotype [28]. Nucleotide variations relative to the ancestral sequence are shown. The *CCR5* numbering systems used in the literature are shown. Top numbering is based on GenBank accession numbers AF031236 and AF031237; middle numbering is based on GenBank accession number U95626; bottom numbering is the numbering system in which the first nucleotide of the *CCR5* translational start site is designated as+1 and the nucleotide immediately upstream as -1 [28]. ORF, open-reading frame; Wt, wild-type; Δ32, 32-basepair deletion.

Human genomic DNA was extracted from whole blood using the Qiagen QIAamp DNA minikit (Qiagen, Hilden, Germany). Polymorphisms in the promoter regions of CCR5 (A29G, G208T, G303A, C630T, T627C, A676G, C927T), the coding regions of *CCR5* (*CCR5* Δ32) and *CCR2*–V64I (G190A) were determined by Taqman Allelic Discrimination assay (AppliedBiosystems, California, US) or PCR-RFLP assays as described previously [9], [27]. **Figure 1** shows the prevailing numbering systems used for *CCR5* polymorphisms and the evolutionary-based classification of *CCR5* haplotypes (HHA to HHG*2) as described previously [28]. The copy number of *CCL3L1* was available from a previous study [7].

### Statistical Analyses

The outcomes were HCV and HIV serostatus, and the explanatory variables were *CCR5* haplotypes or genotypes. Differences in the distribution of *CCR5* haplotypes between study groups were compared by Chi-square or Fisher exact tests. Uni- and multivariate logistic regression models were used to determine the associations between genotype and HCV and/or HIV before and after adjustment to co-variates. The co-variates were age, gender, HBV infection, length of IVDU (in years), *CCL3L1* copy number, and where appropriate concomitant HCV or HIV infection status.

## Results

### Study Population

**Table 1** shows the distribution of HIV, HCV and HBV serostatus among 373 IDUs. Of these, 14% (n = 53) were seronegative for HIV, HCV and HBV, whereas 27% (n = 99) and 7% (n = 27) were seropositive for only HCV and HIV, respectively. 35% (n = 130) were dually infected with HIV and HCV, and 12% (n = 44) were seropositive for HIV, HCV and HBV. The proportion of subjects with HBV and HIV (n = 4, 1%) or monoinfection with HBV (n = 1) was low.

**Table 1:** HIV, HCV and HBV serostatus among 373 IDUs from Estonia in 2006–2007*.

HIV	HCV	HBV	n (%)
+	+	+	44 (12%)
+	+	–	130 (35%)
+	−	+	4 (1%)
−	+	+	8 (2%)
+	−	−	27 (7%)
−	+	−	99 (27%)
−	−	+	1 (0%)
−	−	−	53 (14%)

* HBV serostatus was unknown for 7 individuals.

**Table 2** shows the univiarate associations for risk of HCV and HIV in the study participants. The likelihood (odds) of HCV seropositivity was 3.04 fold (95% confidence interval (CI) = 1.85–5.01) or 3.63–fold (95% CI = 1.40–9.42) respectively higher in those who were HIV or HBV seropositive (**Table 2**). Each additional year of IVDU increased the risk of HCV seropositivity by 1.23 fold (95% CI = 1.12–1.35). Similarly, subjects with HCV or HBV seropositivity were 3.04- and 5.16-fold, respectively, more likely to be HIV seropositive and each additional year of IVDU increased the risk of HIV seropositivity by 1.08 fold. The *CCL3L1* copy number was considered as a co-variate in the subsequent multivariate analyses, and its associations with HIV and HCV seropositivity were consistent with those reported previously [7]. Age and gender did not associate with either HCV or HIV serostatus.

**Table 2:** Factors influencing HCV and HIV serostatus by univariate analyses.

Variable	Comparison	Outcome: HCV serostatus	Outcome: HIV serostatus
		OR; 95% CI; *P*	OR; 95% CI; *P*
Gender	Female vs Male	0.63; 0.34–1.17; 0.142	1.16; 0.66–2.05; 0.609
Age (years)	Years#	1.05; 0.99–1.11; 0.105	1.02; 0.98–1.07; 0.357
HCV status	HCV+vs HCV−	n/a	3.04; 1.85–5.01; 1.70×10^−5^
HIV status	HIV+vs HIV−	3.04; 1.85–5.01; 1.70×10^−5^	n/a
HBV status	HBV+vs HBV−	3.63; 1.40–9.42; 0.008	5.16; 2.45–10.89; 2.19×10^−5^
IVDU (years)	years*	1.23; 1.12–1.35; 7.51×10^−5^	1.08; 1.02–1.16; 0.015
*CCL3L1* copy	>2 vs 0–2&	1.16; 0.63–2.13; 0.646	0.48; 0.29–0.81; 0.005

# The OR was estimated by every increased year of age;

* The OR was estimated by every increased year of IVDU use;

& The median of *CCL3L1* copy number is 2 copies in our studied IDUs.

### The Distribution of *CCR5* Haplotypes and Haplotype Pairs

**Figure 2:**
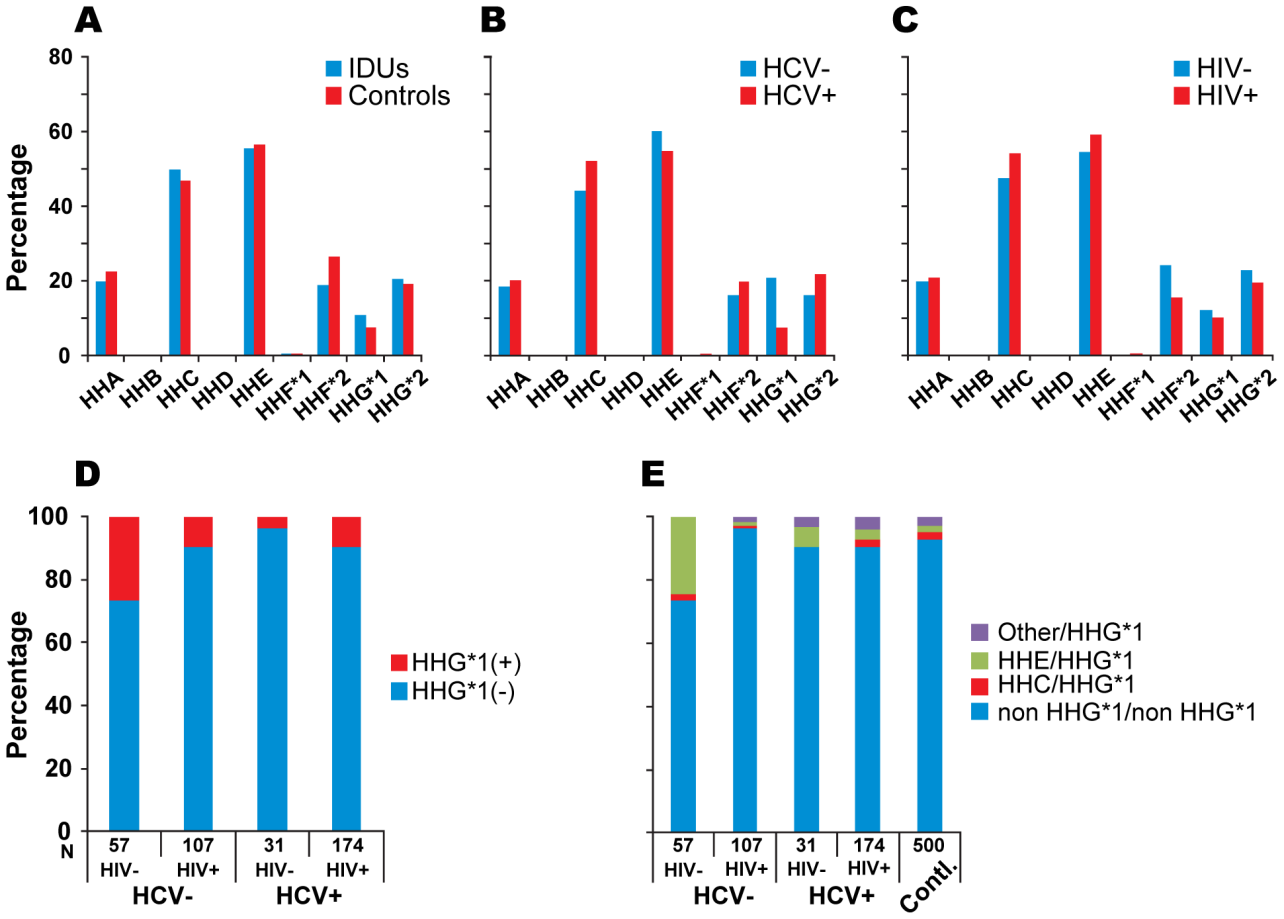
The distribution of *CCR5* haplotypes among IDUs and blood donors. *CCR5* haplotype frequency among (**A**) IDUs vs. blood donors, (**B**) HCV+vs. HCV- IDUs, and (**C**) HIV- vs. HIV+IDUs. HHB and HHD are absent in the study populations. Frequency of (**D**) *CCR5* HHG*1 haplotype and (**E**) *CCR5* HHG*1-containing genotypes in IDUs by HCV and HIV serostatus.

Complete *CCR5* genotype data was available from two study populations - 369 IDUs and 500 seronegative blood donors. All SNPs were in Hardy-Weinberg equilibrium in both study groups. The most frequent *CCR5* haplotypes in both study groups were HHE and HHC; approximately half of the IDUs and blood donors possessed these haplotypes (**Figure 2A**). Consistent with their African-specific distribution [8], [28], *CCR5*-HHB and -HHD haplotypes were not found in this Caucasian population (**Figure 2A**).

### Associations of *CCR5* Haplotypes with HCV Serostatus

To investigate whether *CCR5* haplotypes are associated with HCV seropositivity, we compared the prevalence of *CCR5* haplotypes between HCV seropositive (HCV+) and HCV seronegative (HCV-) IDUs, before and after accounting for concomitant HIV serostatus. The *CCR5* HHG*1 haplotype was over-represented in HCV- compared with HCV+subjects (20.7% vs. 7.5%, respectively; p<0.001) (**Figure 2B**). In a multivariate logistic regression model that included all *CCR5* haplotypes (HHA to HHG*2), only the HHG*1 haplotype associated significantly with HCV seropositivity (OR = 0.37; 95% CI = 0.17–0.84; P = 0.017). We then determined the association of HHG*1 with HCV serostatus after controlling for HIV and HBV serostatus, as well as duration of IVDU. In this multivariate model, possession of the CCR5-HHG*1 haplotype associated with 93% lower risk of HCV seropositivity compared with those lacking this haplotype (OR = 0.07, 95% CI = 0.03–0.20, P<0.0001).

### Associations of *CCR5* Haplotypes with HIV Serostatus

When using HIV as an outcome, we observed that HHF*2 was over-presented in HIV-negative (HIV-) IDUs compared with HIV-positive (HIV+) IDUs (**Figure 2C**). The likelihood of bearing an HHF*2 haplotype was 43% lower in HIV- compared with HIV+IDUs (OR = 0.57, 95% CI = 0.34–0.98, P = 0.041). These data were in agreement with the published literature suggesting that the HHF*2 associates with a lower risk of acquiring HIV infection [8], [29]. However, in multivariate logistic regression models after controlling for co-variates (HCV and HBV serostatus, length of IVDU and *CCL3L1* copy number) or the other *CCR5* haplotypes, the association of HHF*2 with a lower risk of HIV seropositivity was not evident (OR = 0.65; 95% CI = 0.34–1.24; P = 0.19). All other haplotypes were equally represented among HIV+and HIV- IDUs.

### Associations of CCR5-HHG*1 with HCV after Accounting for HIV Co-infection

Although we found that HHG*1 associated with a lower rate of HCV seropostivity after accounting for multiple comparisons and non-genetic co-variates, a potential confounder was that a significant proportion of the HCV+individuals were co-infected with HIV (35%), and conversely, 8% of HCV- subjects were HIV-positive (**Table 1**). To account for this potential confounder, we defined the associations of HHG*1 according to HCV and HIV serostatus. HHG*1 haplotype was significantly overrepresented in subjects who were both HIV and HCV seronegative compared with subjects who were HCV+only, HIV+only or were HCV+and HIV+(**Figure 2D**). After controlling for co-variates, compared with individuals who were HCV−/HIV- (reference category), the likelihood of possessing a HHG*1-containing genotype was lower by 92% (OR = 0.08; 95% CI = 0.02–0.29) and 82% (OR = 0.18; 95% CI = 0.06–0.54) in participants who were HCV+/HIV-, and HCV+/HIV+, respectively; the association in participants who were HCV−/HIV+was not significant (OR = 0.43; 95% CI = 0.10–1.76) (**Table 3** **,** models 1 to 3).

**Table 3:** Association of *CCR5* HHG*1 with HCV or HIV serostatus.

Model	Study groups	Unadjusted	Adjusted#
		OR; 95% CI; P	OR; 95% CI; P
HHG*1 vs non-HHG*1			
1	HCV+HIV− vs. HCV−HIV−	0.11; 0.03–0.35; 2.0×10^−5^	0.08; 0.02–0.29; 1.89×10^−4^
2	HCV−HIV+vs. HCV−HIV−	0.29; 0.08–1.12; 0.077	0.43; 0.10–1.76; 0.242
3	HCV+HIV+vs. HCV−HIV−	0.29; 0.13–0.64; 0.002	0.18; 0.06–0.54; 0.003
HHE/HHG*1 vs non−HHG*1/non−HHG*1			
4	HCV+HIV− vs. HCV−HIV−	0.03; 0.00–0.23; 0.001	0.02; 0.00–0.20; 0.001
5	HCV−HIV+vs. HCV−HIV−	0.21; 0.04–1.01; 0.055	0.30; 0.06–1.58; 0.161
6	HCV+HIV+vs. HCV−HIV−	0.11; 0.04–0.30; 2.76×10^−5^	0.07; 0.01–0.32; 0.001

# Co-variates: HBV serostatus, *CCL3L1* copy number, IVDU duration and HBV serostatus.

We next determined which specific HHG*1-containg genotype contributed to the reduced seropositivity of HCV. Among the study participants, the two most common HHG*1-containing genotypes were HHE/HHG*1 and HHC/HHG*1, present in 61% and 16% of the IDUs, respectively. Of these two HHG*1-containing genotypes, HHE/HHG*1 was overrepresented in subjects who were seronegative for both HIV and HCV compared with subjects who were seropositive for HCV and/or HIV (**Figure 2E**). After controlling for co-variates, compared with IDUs who were seronegative for both HIV and HCV, the likelihood of possessing HHE/HHG*1 was lower by 98% and 93% in subjects who were HCV+/HIV- and HCV+/HIV+, respectively; the associations in participants who were HCV−/HIV+was not statistically significant (**Table 3**, models 4 to 6).

On the basis of these findings, we hypothesized that if HHG*1-containing genotypes associated with strong resistance to acquiring HCV or HCV/HIV in IDUs then the prevalence of HHG*1 or HHE/HHG*1 among HCV−/HIV- IDUs should be greatly overrepresented when compared with HCV−/HIV- blood donors. Consistent with this possibility, ∼25% vs.<10% of the HCV−/HIV- IDUs vs. HCV−/HIV- blood donors, respectively, possessed the HHG*1 haplotype (p = 3.3 ×10^−6^) or HHE/HHG*1 haplotype (p = 7.2 ×10^−15^; **Figure 2E**).

### Associations of CCR5-HHG*1 with HCV Serostatus and Potential Confounding Factors

The aforementioned findings suggested *CCR5* HHG*1 haplotype influences HCV serostatus in our studied IDUs. To control for additional confounding factors, we did the following two analyses: First, we conducted a step-wise logistic regression analyses for the association between HHG*1 and HCV serostatus with the following covariates: HIV, and HIV and HBV serostatus, *CCL3L1* copy number, study population (i.e. prisons or syringe-exchange programs), duration of IVDU, age, gender and an interaction term “age×IVDU” (**Table 4**). The interaction term was included because a more pronounced effect of duration of IVDU on HCV seropositivity was found in the younger group (<26 years old, OR = 1.46, p = 7.51×10^−5^) compared to the older group (≥26 years old, OR = 1.19, P = 0.016). Second, we did the same analysis but restricted to the subjects from syringe-exchange program only, restricted to the younger group, and restricted to the older group (data not shown). Our results in each of these analysis indicated that the protective effect of HHG*1 on HCV serostatus persisted after controlling for potential confounding factors.

**Table 4:** Association of *CCR5* HHG*1 with HCV serostatus in univariate and stepwise multivariate logistic regression model.

Models	n	OR	95% CI	P-value	
Univariate model					
HHG(+) vs HHG(−)	368	0.31	0.16–0.61	8.79×10^−4^	
**Multivariate models**					
Adjusted for HIV serostatus					
HHG(+) vs HHG(−)	368	0.30	0.15–0.63	1.35×10^−3^	
Adjusted for HIV and HBV serostatus,					
HHG(+) vs HHG(−)	362	0.29	0.14–0.61	1.17×10^−3^	
Adjusted for HIV, HBV serostatus and *CCL3L1* copy number*					
HHG(+) vs HHG(−)	362	0.30	0.14–0.64	0.001	
Adjusted for HIV, HBV serostatus, *CCL3L1* copy number*, and study population#					
HHG(+) vs HHG(−)	343	0.21	0.10–0.45	8.32×10^−5^	
Adjusted for HIV, HBV serostatus, *CCL3L1* copy number*, and duration of IVDU&					
HHG(+) vs HHG(−)	228	0.08	0.03–0.421	9.47×10^−7^	
Adjusted for HIV, HBV serostatus, *CCL3L1* copy number*, duration of IVDU&, age and gender					
HHG(+) vs HHG(−)	227	0.07	0.03–0.19	5.77×10^−7^	
Adjusted for HIV, HBV serostatus, *CCL3L1* copy number*, duration of IVDU&, age, gender and IVDU×age					
HHG(+) vs HHG(−)	227	0.06	0.02–0.17	2.07×10^−7^	

* Dichotomized by the median of 2 *CCL3L1* copies;

# Syringe exchange program or Prisoners;

& Each additional year of IVDU use.

## Discussion

**Figure 3:**
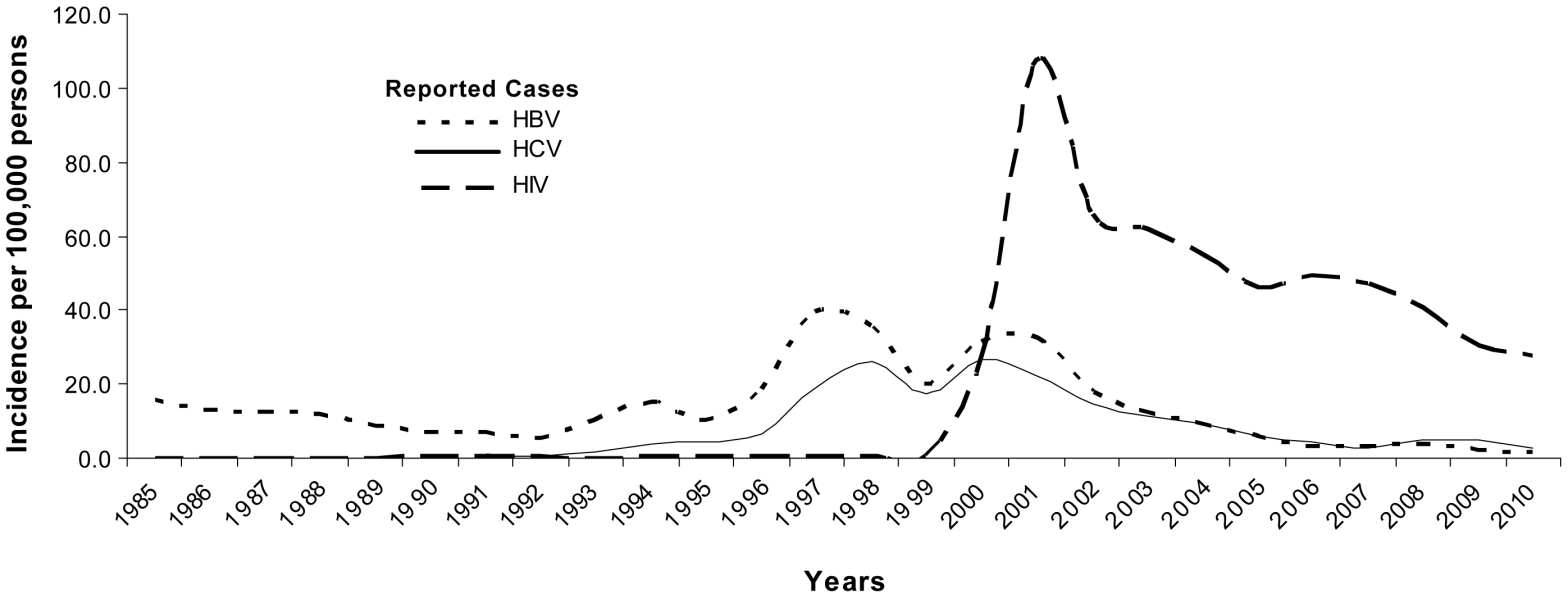
Prevalence of HIV, HBV and HCV infection in Estonia. Incidence per 100,000 population of HIV (dashed line), HBV (dotted line) and HCV (solid line) infection in Estonia between 1985–2010, as reported by the Estonian Health Board.

We evaluated a group of IDUs from the Estonia in whom nearly 80% of subjects were infected with HCV and/or HIV. The HIV epidemic in the Estonia is unique in that it is a relatively new epidemic with HIV infection rates peaking in 2001–2002 and is localized mainly among IDUs [7], [30] (**Figure 3**). The HIV epidemic was antedated by an increase in infection rates of HBV and HCV by a few years (**Figure 3**). The principal finding of this study is that the CCR5-HHG*1 haplotype associates with strong resistance to HCV infection in a group of IDU’s from Estonia at high risk for HCV and HIV infection. The *CCR5-*HHG*1 haplotype was highly overrepresented among the IDUs that were seronegative for both HCV and HIV comprising nearly 25% of these subjects. In contrast, less than 10% of IDUs who were seropositive for HCV and/or HIV as well as HCV−/HIV- blood donors possessed the HHG*1 haplotype.

A stratified analyses revealed that compared with individuals who resisted acquiring both HCV and HIV (i.e., HCV−/HIV-), subjects who were HCV+/HIV-, HCV−/HIV+, HCV+/HIV+were ∼90%, 57% and 80% less likely to possess the *CCR5*-HHG*1 haplotype, albeit the associations for participants who were HCV−/HIV+did not achieve statistical significance at P<0.05. One interpretation of these findings is that the HHG*1 haplotype mainly influences risk of HCV. This possibility is reinforced by the finding that we and others did not find a substantial difference in the prevalence of the HHG*1 haplotype when comparing HIV+vs. HIV- individuals [9], [12]. However, the subjects who were HCV−/HIV+were also the least represented group in the IDU study population, and consequently, a smaller sample size could have also accounted for the reduced strength of the association. Notwithstanding these caveats, these findings implicate a role for CCR5 in risk of HCV and possibly, co-infection with HIV.

The *CCR5*-HHG*1 haplotype has several noteworthy features that provides insights into a possible basis for the observed associations. Foremost, HHG*1 is the ancestral haplotype upon which the HIV-resisting *CCR5-*Δ*32*-containing HHG*2 haplotype arose [8], [28] (**Figure 1**). Thus, the *CCR5-*HHG*1 haplotype has the same genetic features as the *CCR5-*Δ*32*-containing HHG*2 haplotype except that it lacks the Δ32 mutation. However, HHG*1 differs from HHG*2 in two notable ways. First, HHG*2 is restricted mainly to European populations. In contrast, HHG*1 has a less restricted distribution and is prevalent in both European and non-European populations [8], [9], [12]. Second, heterozygosity and homozygosity of the *CCR5-*Δ*32*-containing HHG*2 haplotype associate with partial vs. complete reductions in CCR5 expression levels, respectively, and these expression patterns in turn contribute to their protective effects in HIV infection [31], [32]. However, in contrast, the influence of HHG*1 with CCR5 expression are unknown.

Although one possibility is that akin to the *CCR5-*Δ*32*-containing HHG*2 haplotype, the associations of the HHG*1 haplotype with reduced HCV and/or HIV risk are related to CCR5 expression. However, another possibility is that the effects are indirect, i.e., related to another gene. We raise this point as the polymorphism in the non-coding region of *CCR5* that is shared by HHG*1 and HHG*2 and which distinguishes it from all the other *CCR5* haplotypes (named as A29G in Figure 1) is in nearly 100% linkage disequilibrium with a polymorphism in a haplotype of *CCRL2* (www.hapmap.org and data not shown) that is ∼31 kb downstream of *CCR5*. Recent studies have demonstrated that this *CCRL2* haplotype associates with multiple diseases [33], [34].

CCR5 is not being expressed on hepatocytes and is not a receptor for HCV entry. It is hypothesized that CCR5 interacts with its ligands to promote the recruitment of Th1 expressing cells into the liver [35], [36]. HCV core protein alters *CCL5* promoter activity [37] resulting in higher levels of CCL5. Increased binding of CCL5 to CCR5 decreases CCR5 surface density due to receptor internalization [38]. These findings together with our results suggest that the chemokine receptor-ligand CCR5-CCL5 system may contribute to acquisition of HCV infection.

Most of the prior studies related to the associations between *CCR5* gene variants and HCV risk have been largely restricted to the CCR5-Δ32 mutation. Woitas *et al*. proposed that *CCR5-*Δ*32* homozygosity (HHG*2/HHG*2) was a susceptibility factor for HCV infection [6]. However, others did not observe this association [17], [23], [39], [40]. In our study population, the *CCR5-*Δ*32/*Δ*32* was not enriched in monoinfected HCV+subjects. The increased frequency of the *CCR5-*Δ*32/*Δ*32* genotype among HCV-infected–HIV-uninfected subjects observed by Woitas et al could have been secondary to the protective effects of this genotype against acquiring HIV infection as their study population comprised mainly of hemophiliacs who were HIV negative [5], [6]. Hence, hemophiliacs have a high risk of both HIV and HCV it is conceivable that the increased prevalence of *CCR5-*Δ*32/*Δ*32* in their study population is due to their HIV-negative status, rather than the HCV-positive status of the monoinfected HCV study group.

As we did not study a seroincident study population, an argument could be made that the overrepresentation of HHG*1 among the HCV−/HIV- IDUs is simply a reflection of an association of HHG*1 with an accelerated HCV or HIV disease course, resulting in the underrepresentation of carriage of HHG*1 among the surviving mono- or dual infected HCV or HIV infected individuals. However, the similarity in the frequency of subjects bearing the HHG*1 haplotype in mono- or dual-infected IDU’s and HIV−/HCV- blood donors (<10% in each) argues against this possibility. Furthermore, the latter observation and the fact that the study participants were all Caucasians from a restricted geographic region in Estonia mitigates against the possibility that the enrichment of HHG*1 among HCV−/HIV- IDUs is due to selective population admixture in this group alone.

Our study has some limitations. First, the duration of IVDU was only known for two thirds of the population and that is mainly from the subjects from the syringe exchange program. However, bearing in mind the similarity of subjects from the two populations in terms of demographic and risk behaviors as well as the short duration of the HIV epidemic in Estonia in general [41], [42], we believe that the duration of IVDU in one population reflects the one in the other. Second, all subjects in the prison cohort were HIV positive and there were more HIV/HCV co-infection among them than in the syringe exchange programs. One of the main reasons is that HIV negative populations cannot be recruited from prisons. However, we emphasize that almost all HIV infected subjects in prisons were infected before they went to prisons. However, by conducting studies in IDUs over several years we have noticed that repeated imprisonment among IDUs is common but short sentences are given. Despite these limitations, we believe that both populations are in essence similar in terms of demographic risk behavior. Thus, the combined analyses of patients from two sources (prisoners plus those from syringe exchange program) do not preclude insights into the relationship of *CCR5* genetics with HIV/HCV serostatus.

In addition, our studied subjects are young with median age of 26 years old. We asked whether our findings are biased due to some individuals might not have enough time of IVDU to get infected of HCV. Although we found a more pronounced effect of duration of IVDU on HCV seropositivity in the younger group compared that in the older group, our mulitivariate analysis in the overall group as well as the stratified analysis in both the younger and older group suggests that confounding of our findings due to age is highly unlikely. Finally, as we have only data for the HCV serostatus (the presence of HCV antibody) but not for active infection (HCV RNA), we cannot evaluate the influence of CCR5 haplotypes on the disease course or HCV clearance. Further studies needed to validate these findings due to the small size of subjects we studied.

In conclusion, we evaluated a large sample size of high-risk subjects and blood donors from a relatively homogenous Caucasian population. This large sample size facilitated categorization of IDU’s into four categories according to their HIV and HCV serostatus, mitigating the potential confounding due to co-infection status. Our study design accounted for three other potential confounders: HBV serostatus, the previously demonstrated strong associations of the *CCL3L1* copy number with protection against HIV infection [7], and length of IVDU. The persistence of the association of *CCR5* HHG*1-containing genotypes with reduced seropositivity of HCV after accounting for these potential confounders strongly suggests a strong role for this genotype, and by extension the *CCR5* locus in HCV infection and possibly HIV infection. Consistent with this possibility, others have shown a role of variations of CCR5 in antiviral responses and chronic HCV infection. However, as noted, given the very high linkage between HHG*1 and a *CCRL2* haplotype that has been shown to influence other infectious and non-infectious diseases, one cannot exclude a possible role for CCRL2 in HCV susceptibility.
